# The Associations between Types of Ambient PM_2.5_ and Under-Five and Maternal Mortality in Africa

**DOI:** 10.3390/ijerph14040359

**Published:** 2017-03-30

**Authors:** Patrick Opiyo Owili, Wei-Hung Lien, Miriam Adoyo Muga, Tang-Huang Lin

**Affiliations:** 1 International PhD Program in Environmental Science & Technology (UST), Institute of Environmental & Occupational Health, National Yang Ming University, Taipei City 112, Taiwan; powili2002@ym.edu.tw; 2 Center for Space and Remote Sensing Research, National Central University, Taoyuan City 320, Taiwan; w.h.lien@hotmail.com; 3 Institute of Community Health and Development, Great Lakes University of Kisumu, Kisumu 40100, Kenya; mugzjnr2000@gmail.com

**Keywords:** ambient air pollution, types of particulate matter, under-five mortality, maternal mortality, Africa

## Abstract

Exploring the effects of different types of PM_2.5_ is necessary to reduce associated deaths, especially in low- and middle-income countries (LMICs). Hence we determined types of ambient PM_2.5_ before exploring their effects on under-five and maternal mortality in Africa. The spectral derivate of aerosol optical depth (AOD) from Moderate Resolution Imaging Spectroradiometer (MODIS) products from 2000 to 2015 were employed to determine the aerosol types before using Generalized Linear and Additive Mixed-Effect models with Poisson link function to explore the associations and penalized spline for dose-response relationships. Four types of PM_2.5_ were identified in terms of mineral dust, anthropogenic pollutant, biomass burning and mixture aerosols. The results demonstrate that biomass PM_2.5_ increased the rate of under-five mortality in Western and Central Africa, each by 2%, and maternal mortality in Central Africa by 19%. Anthropogenic PM_2.5_ increased under-five and maternal deaths in Northern Africa by 5% and 10%, respectively, and maternal deaths by 4% in Eastern Africa. Dust PM_2.5_ increased under-five deaths in Northern, Western, and Central Africa by 3%, 1%, and 10%, respectively. Mixture PM_2.5_ only increased under-five deaths and maternal deaths in Western (incidence rate ratio = 1.01, *p* < 0.10) and Eastern Africa (incidence rate ratio = 1.06, *p* < 0.01), respectively. The findings indicate the types of ambient PM_2.5_ are significantly associated with under-five and maternal mortality in Africa where the exposure level usually exceeds the World Health Organization’s (WHO) standards. Appropriate policy actions on protective and control measures are therefore suggested and should be developed and implemented accordingly.

## 1. Introduction

Ambient and indoor air pollution, particularly from fine mode particulate matters (PM_2.5_, which has a diameter smaller than 2.5 microns), is one of the major concerns of international organizations and governments because of the health effects associated with exposure levels, spatial domain, the age and health of individuals, and pollutant types [1,2]. In 2012, it was estimated that over 11% of global deaths (i.e., 6.5 million) were a result of indoor and ambient air pollution, of which close to 90% of those deaths were in low- and middle-income countries (LMICs) [3]. Moreover, it is also estimated that over 90% of the world’s population live in LMICs where the levels of air quality surpass that of the World Health Organization’s (WHO) air quality standards [3]; that is, the global no-threshold limit for daily (≤25 μg/m^3^) and annual (≤10 μg/m^3^) ambient PM_2.5_ exposure [4]. In recent years, many studies have also focused on the association between PM_2.5_ and all-cause mortality, mainly in high-income countries, with a positive relationship being found [5,6,7,8,9,10]. However, studies on PM_2.5_ and mortality are still limited in low- and middle-income countries (LMICs), where the majority of under-five and maternal preventable deaths occur. This limitation may be due to an increased focus on other causes of death, for example, complications during childbirth, as well as infectious and communicable diseases, which is plausible. Yet, explaining the causes of death is relatively complex owing to the interrelationship that may exist between other potential causes of fatality. Authors have, however, identified that children are very vulnerable to ambient air pollution even at low levels [11]. This raises the question as to whether ambient air pollution is one of the main contributing factors to the high number of under-five and maternal deaths in LMICs. Moreover, because of high spatial and temporal variations in the ambient air quality coupled with seasonal and time changes [12], more studies in different regions on the health effects of short-term and long-term exposure to different types of ambient PM_2.5_ are necessary so as to effect policy changes on regional and/or global air quality standards. An investigation on the effect of ambient PM_2.5_ on under-five and maternal mortality, as in this study, remains significant towards answering such questions and subsequently designing and implementing preventive measures that can lead to improved maternal, newborn, and child health (MNCH) in LMICs.

Ambient air remains a receptor of all kinds of pollutants, both from natural and human activities that are detrimental to population health and ecosystems. Consequently, authors have tried to develop a technique for identifying and categorizing different types of ambient aerosols using spectral aerosol optical depths (AOD) from satellite observations [13]. This development is very important towards exploring the health effects of each type of ambient PM_2.5_, which was utilized in our study. This study, therefore, becomes an entry point and/or a contribution towards discussions related to types of ambient PM_2.5_ and their health effects.

Nevertheless, previous studies have found an association between ambient PM_2.5_ and other negative health effects such as blood pressure, inflammation and oxidative stress, ventricular arrhythmia and thrombotic processes, lung function, and epidemiologic evidence on mortality [6,14,15,16,17,18]. Children, particularly those who are exposed to air pollution, can potentially have chronic low lung function, which is associated with an increased risk of cardiovascular disease and mortality in adulthood [19,20,21]. Most of these studies were conducted in high-income countries, where the majority of the populace have relatively more information on the effects of ambient air pollution, although none of them explored the health effects of different PM_2.5_ types. However, there is still limited literature on the health effects of ambient PM_2.5_ in LMICs that have the highest number of pollution-related deaths, including deaths of children and their mothers [3], and this is in part due to the inadequate data for research in these countries. Our study, therefore, was aimed to determine the types of ambient PM_2.5_ in Africa and then to investigate the change in PM_2.5_ concentrations in recent years. We further explored the associations between different types of ambient PM_2.5_ and under-five and maternal mortality in Africa. This study is significant towards discussions concerning the achievement of Sustainable Development Goals (SDGs), specifically in terms of health-related targets such as the reduction of deaths and illnesses resulting from hazardous substances in the air, water, and soil by 2030 [22].

## 2. Methods

### 2.1. The Spatial Domain

**Figure 1:**
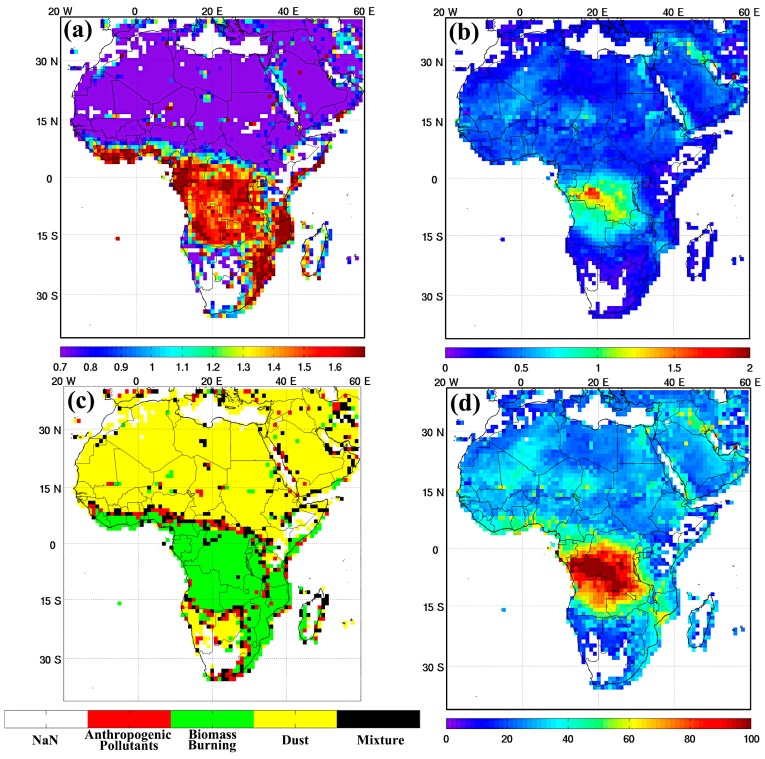
Illustration the MODIS monthly products of (**a**) AOD_660nm_ and (**b**) AE_470_660nm_ in September 2015 (1° × 1°), and (**c**) the spatial distribution of aerosol types including biomass (green), anthropogenic (red), dust (yellow) and mixture (black) and (**d**) the PM_2.5_ concentrations (μg/m^3^).

The spatial domain of our study included 54 countries in Africa that were sub-divided into five geographical sub-regions according to the African Union sub-regions, namely: Northern Africa (i.e., Algeria, Egypt, Libya, Mauritania, Sahrawi Arab Democratic Republic, and Tunisia), Southern Africa (i.e., Angola, Botswana, Lesotho, Malawi, Mozambique, Namibia, South Africa, Swaziland, Zambia, and Zimbabwe), Eastern Africa (i.e., Comoros, Djibouti, Ethiopia, Eritrea, Kenya, Madagascar, Mauritius, Rwanda, Seychelles, Somalia, South Sudan, Sudan, Tanzania, and Uganda), Western Africa (i.e., Benin, Burkina Faso, Cabo Verde, Côte d’Ivoire, Gambia, Ghana, Guinea-Bissau, Guinea, Liberia, Mali, Niger, Nigeria, Senegal, Sierra Leone, and Togo), and Central Africa (i.e., Burundi, Cameroon, Central African Republic, Chad, Congo Republic, DR Congo, Equatorial, Guinea, Gabon, and São Tomé and Príncipe). The contour lines of these countries are also shown in Figure 1. The data for each country included the 16 years period (i.e., 2000 to 2015). The total population-wide data were based on the de facto definition of population, which included all counted residents regardless of their legal status or citizenship, while refugees not permanently settled in their asylum country were included in their country of origin.

### 2.2. Variables

#### 2.2.1. Under-Five and Maternal Mortality Data

We used the annual estimated count data of under-five and maternal deaths for each country (years 2000 to 2015) from the World Bank [23]. Under-five mortality was defined as the number of children who died before reaching the age of five, while maternal death was defined as the number of pregnant women who died from any cause, including those related to pregnancy management.

#### 2.2.2. Types of Ambient PM_2.5_ Assessment

**Figure A1:**
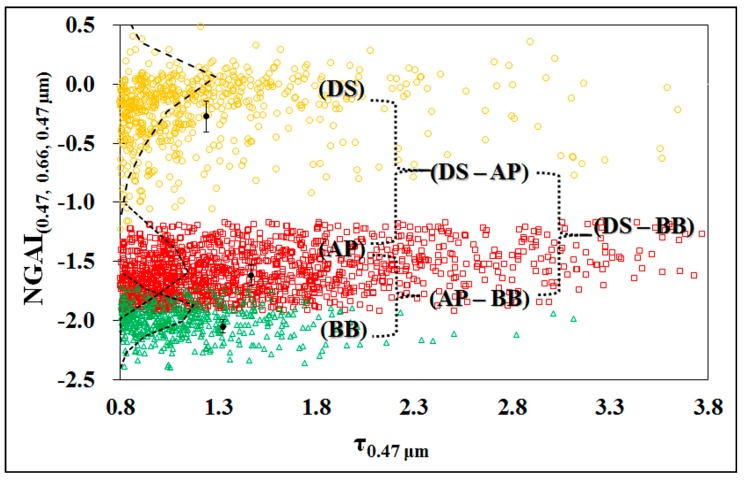
Scatter plots of Normalized Gradient Aerosol Index (NGAI) based on spectral AODs from ground-based measurements for the identification of aerosol type and the dual type mixtures. The dynamic ranges of NGAI_0.47dy_ are −0.27 ± 0.26, −1.62 ± 0.18, −2.05 ± 0.11 for DS, AP and BB aerosols, respectively.

We used the annual mean ambient PM_2.5_ concentrations for 16 years (i.e., 2000–2015) derived from the Moderate Resolution Imaging Spectroradiometer (MODIS) monthly aerosol optical depth products (MOD04/Terra and MYD04/Aqua) over the study area [24]. To determine the types of ambient PM_2.5_, derivations of spectral aerosol optical depth (AOD) were applied to discriminate aerosol categories between mineral dust (DS), anthropogenic pollutant (AP), and biomass burning (BB), a method recently published elsewhere [13]. The temporal and spatial exposures of different aerosol types were thus generated (see also the Appendix A). For the AOD-PM_2.5_ relationship, it is important to identify the dissimilarity between aerosol types due to the optical properties of absorption and scattering. Therefore, the PM_2.5_ concentration was computed according to the relationship of the AOD-PM_2.5_ for each aerosol type [25]. An example of the types of PM_2.5_ concentration from the MODIS AOD product is illustrated in Figure 1; the types of aerosols and mixtures were included. The monthly mean AOD_660nm_ (Figure 1a) and mean AE_470_660nm_ (Figure 1b) of the MODIS products in September 2015 are displayed. In the aerosol type identification (Figure 1c), the high values of AOD (≥0.8) and AE (≥1.4) indicated the frequent emission of BB (i.e., green color), as seen in Central Africa during the month of September 2015. This result is quite consistent with a previous investigation, which indicated that BB is a major source of aerosol that normally takes place during the dry season [26]. Thus, the PM_2.5_ concentrations (μg/m^3^) were derived accordingly (Figure 1d). The types of PM_2.5_ (μg/m^3^) were derived using Equations (1) to (3) as below [25]:

(1) $$PM_{2.5}^{DS}=52.8\times {\mathrm{AOD}}_{\mathrm{660nm}}+9.68$$

(2) $$PM_{2.5}^{AP}=62.4\times {\mathrm{AOD}}_{\mathrm{660nm}}+12.4$$

(3) $$PM_{2.5}^{BB}=98.3\times {\mathrm{AOD}}_{\mathrm{660nm}}+15.4$$

where the $PM_{2.5}^{Type}$ expresses the PM_2.5_ value derived for aerosol types (DS, AP, or BB). In this study, the mixture type considered all three kinds of mixture types (DS-AP, AP-BB, and DS-BB; see also the Figure A1 in Appendix A for the mixture points). The PM_2.5_ concentrations with mixture type were computed by an AOD fraction, which derived the AOD value and PM_2.5_ of each aerosol type from the three kinds of mixture types [13], after which each PM_2.5_ value was summed up to get the total PM_2.5_ concentration of each mixture type.

#### 2.2.3. Covariates

Several covariates were used in this study, which included the country, year, total population, urban population, female population, employed population, number of deaths as result of AIDS and tuberculosis, number of undernourished people, prevalence of anemia among pregnant women, and the annual mean temperature. We considered data from the most recent years in our study (i.e., from 2000 to 2015). The total population included all residents irrespective of citizenship or legal status, while the urban population was based on the national statistical office’s definition of the number of people who dwell in urban areas, and the female population included all female residents regardless of citizenship or legal status. The employed population was defined as the percentage of a country’s population aged 15 years and above that is employed. Other deaths, such as AIDS deaths, were estimated as the number of adults and children who died due to AIDS-related causes, while the tuberculosis death rate was expressed as per 100,000 population, which was estimated among the HIV-negative population. The number of people who were undernourished was defined as those whose food intake was insufficient to meet dietary energy requirements continuously. Anemia among pregnant women was defined as the proportion of pregnant women whose hemoglobin level is less than 110 grams per liter (g/L) at sea level. The annual mean temperature was expressed as degrees Celsius and was aggregated to include both the country and basin levels.

### 2.3. Statistical Analysis

Before conducting the analysis, we used an Expectation Maximization (EM) algorithm technique to impute the missing data on the variables that had a few missing data; this approach is the most modern technique that caters for Missing at Random (MAR) and Missing Completely at Random (MCAR) assumptions and gives consistent estimates, even with up to 50% of missing data [27]. The 54 countries were expected to have annual data of each variable for the 16 years period (i.e., 864 total cases), however, it was not the case due to missing cases in the following variables: biomass PM_2.5_ (21); anthropogenic PM_2.5_ (126); dust PM_2.5_ (84); mixture PM_2.5_ (54); total population (54); urban population (54); female population (54); employed population (114); AIDS deaths (99); tuberculosis deaths (65); undernourished population (319); anemia in pregnancy (228); annual temperature (178); and maternal mortality (16). In all the indicators used, only undernourished population had highest number of missing data but all were still less than 50% (i.e., range of the missing data = 1.9%–36.9%).

The monthly ambient PM_2.5_ concentrations were then used to determine the annual mean PM_2.5_ concentrations, before plotting the trends of under-five deaths, maternal deaths, and types of PM_2.5_ concentration by sub-regions of Africa. Our descriptive statistics reported the types of PM_2.5_ concentration on a moving average, while the annual mean PM_2.5_ concentration was used to estimate the association with under-five and maternal mortality.

Two regression approaches, parametric and nonparametric, were employed in our analysis. In the parametric approach, a Generalized Linear Mixed-Effect Model with natural cubic splines (GLMM+NS), spatial covariance structure, and Poisson link function was used by applying the Penalized Quasi-Likelihood (PQL) estimation approach to account for the repeated measurement effect [28]. This analytical procedure (i.e., GLMM) allowed us to specify the random effect, the covariance structure, and the distribution of the dependent variable; it is a useful approach in parametric regression when the exposure or the outcome is correlated and over-dispersed as it provides a unified mixed effects likelihood framework [29]. The spatial covariance structure accounted for spatial correlation in the ambient air across different regions and times. On the other hand, the Generalized Additive Mixed-Effect Model (GAMM) was used with a smoothing function and a random effect model to estimate the nonlinear relationship in the data. The GAMM has a relaxed assumption on the independence of the observation, which allows for fitting a nonparametric relationship. We built two models, with the first model unadjusted while the second one was adjusted for all the variables. The analysis was stratified according to the geographical sub-regions of Africa.

Finally, the GAMM with a penalized spline smoothing function, a random intercept and spatial covariance structure, and a Poisson link function was used to assess the PM_2.5_ mortality dose-response relationship. The degrees of freedom of the penalized splines for PM_2.5_ mortality relationship were estimated using generalized cross-validation (GCV). The GAMM penalized spline accounts for the correlation and over-dispersion by using additive nonparametric approaches, and allows for the random effects in the additive predictor; it is also used in the crossed and nested designs and in the analysis of hierarchical, clustered, and spatial data [29]. Stata version 13.1 (StataCorp, College Station, Texas, TX, USA) and R version 3.2.2 (R Foundation for Statistical Computing, Vienna, Austria) software were used in our analyses.

## 3. Results

**Figure 2:**
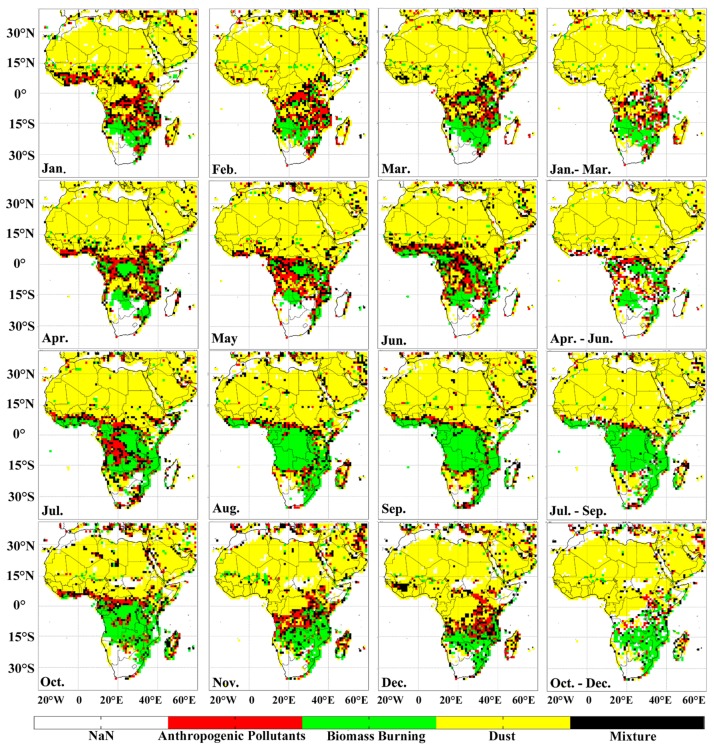
The spatial distribution of type aerosols from January to December 2015. The colors represent different PM_2.5_ types: green (biomass burning), red (anthropogenic), yellow (dust), and black (mixture).

Table 1 presents the mean difference of long-term under-five and maternal deaths, aerosol optical depth, types of PM_2.5_, and population characteristics of the sub-regions of Africa. All the indicators were statistically significant at *p* < 0.001, except for a proportion of the female population that was significant at *p* = 0.045, while the undernourished population was not significant (*p* = 0.129). The moving averages of almost all the types of PM_2.5_ concentration were slightly higher after the first three months in almost all of Africa’s sub-regions, while some sub-regions had a higher PM_2.5_ average in the first tri-month (i.e., dust concentration in the Western and Central sub-regions). The monthly spatial distribution of types of aerosols for the year 2015 is shown in Figure 2.

**Figure 3:**
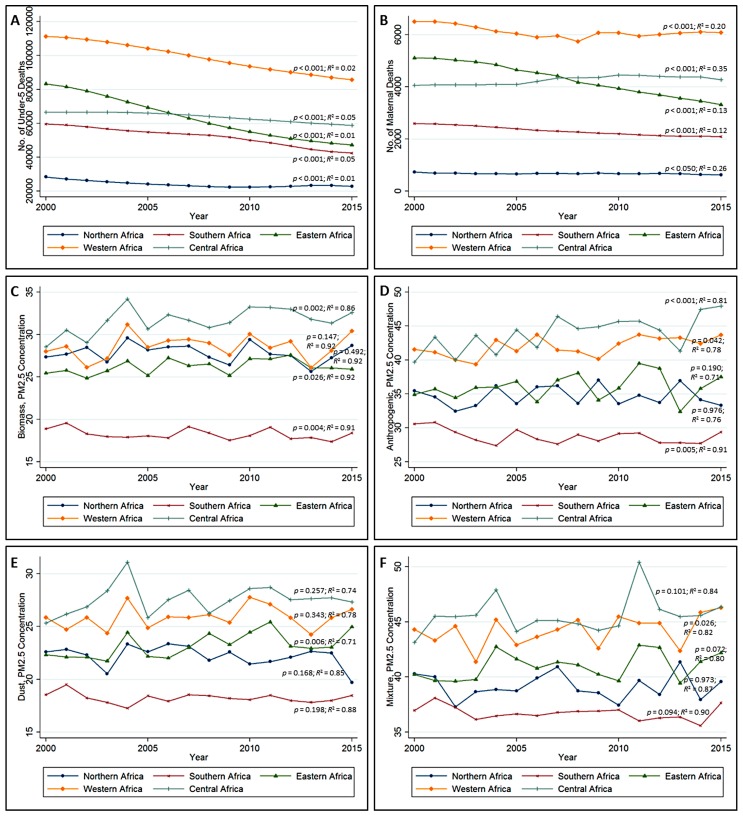
The annual mean trend of (**A**) under-5 deaths, (**B**) maternal deaths and (**C**) the PM_2.5_ concentrations of biomass, (**D**) anthropogenic, (**E**) dust and (**F**) mixture by sub-regions of Africa. The GLMM with random intercept (country and year) was used to obtain the p-values of the change across the years. The GLMM with random intercept (country and year) was used to obtain the *p*-values and the *R*^2^*.*

Figure 3 presents the annual mean trend of under-five deaths, maternal deaths, and types of PM_2.5_ concentration by sub-regions of Africa. The GLMM results of change in the under-five mortality (Figure 3A) and maternal mortality (Figure 3B) trend were significant in all the sub-regions (*p* < 0.001) with greatest decline in under-five deaths realized in Eastern and Western Africa. Nevertheless, the annual mean of both under-five and maternal deaths remained high and low in Western Africa and Northern Africa, respectively. The change in the biomass of PM_2.5_ concentration over time (Figure 3C) in all the sub-regions was statistically significant, except for the sub-regions of Western Africa (*p* = 0.147) and Northern Africa (*p* = 0.492), while only Eastern Africa had a significant change in its dust particulate concentration over time at *p* = 0.006 (Figure 3E). However, for anthropogenic PM_2.5_ concentrations (Figure 3D) only Northern Africa (*p* = 0.976) and Eastern Africa (*p* = 0.190) were not statistically significant. The conditional *R*^2^ which explains the variance of both fixed and random effect is reported, unlike the marginal *R*^2^ which explains only the variance of a fixed effect [30].

The change in particulate concentrations of mixture was marginally significant in almost all the sub-regions, except for Western Africa, which was statistically significant at *p* = 0.026, and Northern Africa, which was not significant (*p* = 0.973). The conditional *R*^2^ of the pollutants were very high because of the high variation existing between countries and across the years.

Table 2 presents the unadjusted incidence rate ratios (IRRs) and 95% confidence intervals (CIs) of under-five and maternal deaths for the moving average of PM_2.5_ concentrations. Annual PM_2.5_ concentrations of biomass were associated with an increased incidence rate of under-five deaths (IRR = 1.02; 95% CI, 1.01–1.03) and maternal deaths (IRR = 1.07; 95% CI, 1.03–1.10), while that of the anthropogenic particulate was only associated with maternal deaths (IRR = 1.03; 95% CI, 1.01–1.04).

The under-five children who were exposed to ambient dust particulates for 12 months had an increased incidence of death by 3%, while that of mothers decreased (IRR = 0.91; 95% CI, 0.90–0.92). Moreover, the under-five children and mothers who had an annual exposure to mixture particulates had an increased mortality incidence of 1.01 (95% CI, 1.01–1.02) and 1.07 (95% CI, 1.06–1.09), respectively. As the year increased, there was a decrease in the incidence of under-five and maternal mortality by 34% and 19%, respectively.

A one unit increase in the urban population increased the death rate of only the under-five children by 2%. However, an increase in the proportion of the employed population by one unit increased the mortality incidence of under-five children and mothers by 10% and 36%, respectively. Additionally, an increase in the tuberculosis death rate increased the incidence of under-five deaths and maternal deaths by 11% and 37%, respectively. However, as the proportion of pregnant women with anemia increased by one unit, the incidence of under-five deaths only increased by 1%.

Table 3 presents the adjusted results of the association between the concentration of ambient particulate types and under-five and maternal deaths by sub-regions of Africa. In general, the GLMM result indicated that the incidence of maternal death in Africa decreases with an increase in biomass particulates by 6%. A stratified analysis by sub-regions indicated under-five deaths and maternal deaths had an incidence rate of 2%, each with a one unit increase in biomass concentration. On the contrary, an increase in biomass concentration reduced the mortality incidence of under-five children in Northern Africa and Eastern Africa by 10% and 2%, respectively, while mothers in Central Africa had the highest incidence rate of death from biomass (IRR = 1.19; 95% CI, 1.15–1.25). The result of the nonlinear relationship of biomass PM_2.5_ also indicated a positive association with under-five deaths (*β* = 0.02, *p* < 0.001) and maternal deaths (*β* = 0.06, *p* < 0.001) in Africa.

The linear relationship result of the anthropogenic concentration indicated an increase in the incidence of under-five children deaths and maternal deaths in Africa by 1% and 3%, respectively. The nonlinear point estimated between anthropogenic and maternal mortality in Africa was also as high as 4%. In Northern Africa, however, an increase in the anthropogenic concentration increased the mortality incidence of under-five children and mothers with IRR = 1.05 (95% CI, 1.02–1.05) and IRR = 1.10 (95% CI, 1.00–1.20), respectively, while in Eastern Africa it only increased the incidence of maternal death by 4%. The nonlinear association of anthropogenic PM_2.5_ also showed a positive relationship with under-five deaths (*β* = 0.002, *p* < 0.001) and maternal deaths (*β* = 0.03, *p* < 0.001) in Western Africa.

Even though the GLMM result of Africa indicated a negative relationship between dust PM_2.5_ and under-five deaths, the sub-regions of North Africa (IRR = 1.03; 95% CI, 1.01–1.06), Western Africa (IRR = 1.01; 95% CI, 1.01–1.01), and Central Africa (IRR = 1.10; 95% CI, 1.09–1.10) were positive. The incidence of maternal deaths as a result of dust PM_2.5_ decreased in Eastern Africa (5%), Western Africa (6%), and Central Africa (4%), and had a similar direction as that of the nonlinear relationship. However, the mixture PM_2.5_ had the greatest effect on maternal mortality incidence in Eastern Africa at IRR = 1.06 (95% CI, 1.02–1.09; *β* = 0.04).

**Figure 4:**
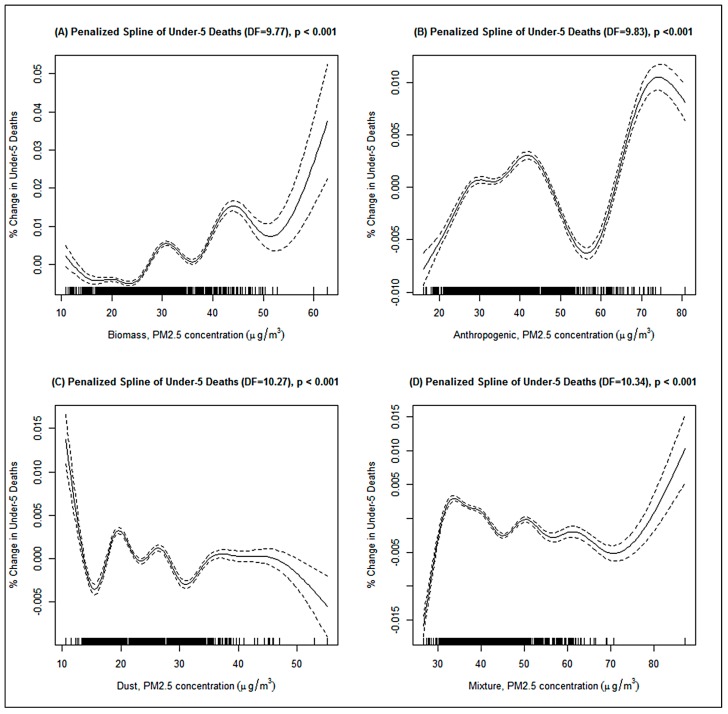
GAMM-estimated relationship between long-term PM_2.5_ concentration and under-5 mortality in Africa. (**A**) Biomass PM_2.5_; (**B**) Anthropogenic PM_2.5_; (**C**) Dust PM_2.5_; and (**D**) Mixture PM_2.5_. Adjusted for country, year, total population, urban population, female population, employed population, AIDS death, tuberculosis death rate, undernourished population, and temperature. The continuous line shows the dose-response relationship while the broken line corresponds to 95% confidence interval.

Figure 4 shows the penalized spline for long-term exposure to PM_2.5_ concentration by type in Africa. We found a nonlinear relationship between the types of PM_2.5_ and deaths of under-five children. All the penalized splines were statistically significant at *p* < 0.001. There was a small effect of biomass PM_2.5_ (Figure 4A) and mixture PM_2.5_ (Figure 4D) on under-five mortality, with a slight increase in death just before a concentration of 30 μg/m^3^ was reached, while for the anthropogenic PM_2.5_ there was evidence of a smaller effect above a concentration of 35 μg/m^3^ (Figure 4B). Dust PM_2.5_ increased under-five mortality slightly below a concentration of 20 μg/m^3^. All the degrees of freedom were above 9.77 and the spline shapes were different.

**Figure 5:**
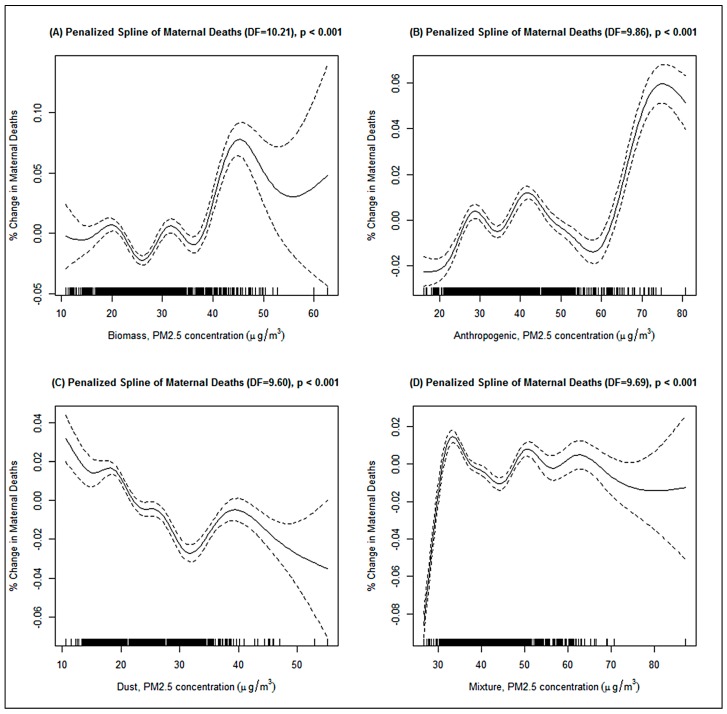
GAMM-estimated relationship between long-term PM_2.5_ concentration and maternal mortality in Africa In terms of (**A**) Biomass, (**B**) anthropogenic, (**C**) dust, and (**D**) mixture PM_2.5_ concentration. Adjusted for country, year, total population, urban population, female population, employed population, HIV/AIDS death, tuberculosis death rate, undernourished population, and temperature. The continuous line shows the dose-response relationship while the broken line corresponds to 95% confidence interval.

Figure 5 shows the penalized spline of the nonlinear relationship between the types of PM_2.5_ concentrations and maternal mortality in Africa, of which there was a nonlinear association. The relationship between the biomass PM_2.5_ and maternal deaths was positive at concentrations slightly above 38 μg/m^3^ (Figure 5A); however, there was a wide confidence interval after 45 μg/m^3^. A slight increase in maternal deaths was also observed above a concentration of 38 μg/m^3^ for the anthropogenic particulate (Figure 5B) and for mixture particulate it was at 30 μg/m^3^ (Figure 5D). The relationship between dust PM_2.5_ and maternal mortality was negative at concentrations of about 25 μg/m^3^ (Figure 5C). All the degrees of freedom were slightly different, but above 9.60. The shapes were also different.

## 4. Discussion

In this study, we determined the types of ambient PM_2.5_ in Africa and investigated the change over time, including the change in under-five and maternal mortality, and subsequently we explored the associations between different types of ambient PM_2.5_ and under-five and maternal mortality. Four types of ambient PM_2.5_ were identified (BB, AP, DS, and mixture), and we found that even though there was a decline in the under-five and maternal deaths over the past decade, different types of ambient PM_2.5_ concentrations were still associated with under-five and maternal deaths in almost all the sub-regions of Africa. The trend also indicated that PM_2.5_ concentrations remained high in Central and Western Africa and low in the Southern Africa sub-region across the study periods. Our penalized spline model also revealed a positive dose–response relationship between types of PM_2.5_ and under-five and maternal deaths; chronic exposure to PM_2.5_ concentrations above 30 μg/m^3^—higher than WHO’s daily (25 μg/m^3^) and annual (10 μg/m^3^) exposure limits—was associated with an increase of death in Africa [4].

In the linear model, we found that biomass PM_2.5_ increased under-five deaths in Central Africa (IRR = 1.02; 95% CI, 1.02–1.03) and Western Africa (IRR = 1.02; 95% CI, 1.01–1.04), while in the nonparametric relationship a positive association was only observed in Southern Africa (*β* = 0.03, *p* < 0.01) and the entire African continent (*β* = 0.02, *p* < 0.001). For maternal mortality, biomass burning only increased the rate of death in Central Africa (IRR = 1.19; 95% CI, 1.15–1.23), which was the highest effect size in a linear relationship, and the rate of death in the whole of Africa (*β* = 0.06, *p* < 0.01, in a nonlinear association). Our finding was consistent with previous findings on PM_2.5_ and mortality [5,6,18]. However, most previous studies did not consider the effect of PM_2.5_ type on under-five and maternal deaths, which can lead to an underestimation or overestimation of the effect; it may, for instance, be assumed that biomass, the main pollutant in Central Africa, is the major cause of under-five deaths rather than other pollutants, and yet our study found that these children had a higher rate of death from dust PM_2.5_ (10%) than biomass PM_2.5_ (2%). Hence, this leads to such questions as: Where does the dust PM_2.5_ in Central Africa come from? Is it blown by wind from Northern Africa after the settling of larger particles? Does the population in Central Africa know how to handle the dust problem as much as the people in Northern Africa? These questions among others only need to be explored further. However, ambient air pollution affects everyone alike and several other health problems have also been associated with it in high-income countries such as heart disease, stroke, chronic and acute respiratory diseases, and lung cancer [31], which may lead to a higher death rate within a country.

We also found that long-term exposure to anthropogenic PM_2.5_ substances majorly increased the under-five and maternal death rates in Northern Africa by 5% and 10%, respectively. Furthermore, it increased maternal deaths in Eastern Africa by 4%. Nevertheless, anthropogenic pollutants increased the under-five deaths and maternal death rates in the entire African continent by 1% and 3%, respectively, and these pollutants, such as NO_2_ and SO_2_, are largely a result of combustion processes due to human activities (e.g., burning fossil fuels for motor vehicles, power generation, and heating). Epidemiological studies have also shown that these anthropogenic pollutants can biologically affect both humans and ecosystems alike; these health problems are associated with the respiratory system, lung function, cardiac disease, and mortality (in humans), as well as reproductive function, offspring behavior, and bone structure in animals [31,32].

Generally, dust particulates did not increase under-five deaths or maternal deaths in the entire continent of Africa. Only the under-five children in Northern Africa, Western Africa, and Central Africa were highly susceptible to death from dust PM_2.5_ by 3%, 1%, and 10%, respectively, in the linear model. As much as dust particles are a problem in Northern Africa, the effect of smaller particles should be one of the greatest concerns in terms of the health and wellbeing of under-five children in Northern, Western, and Central Africa. Central Africa had the largest effect size from dust particulates. However, the rate of maternal death did not increase with PM_2.5_ in all the sub-regions.

We also found that the mixture PM_2.5_ increased the rate of under-five deaths only in Western Africa by 1%, and for maternal deaths, the rate increased only in Eastern Africa by 6%. Our study’s findings demonstrated that by exploring the effect of each type of PM_2.5_, researchers can determine the effects of the different pollutants suspended in the ambient air and subsequently allow for the development of policy and the implementation of appropriate control and preventive measures in each country.

The WHO’s global air quality no-threshold level was set at 25 μg/m^3^ (for acute exposure) and 10 μg/m^3^ (for chronic exposure), which is reasonable, and yet our study found that the annual mean levels of different types of PM_2.5_ exceeded the WHO’s standards across the 16-year period. Moreover, our dose-response relationship curve showed a no-threshold limit concentration of about ≥30 μg/m^3^ for chronic exposure to biomass, anthropogenic, and mixed particulates in Africa. On the contrary, both under-five and maternal death rates were high with low concentrations of dust PM_2.5_ (i.e., approximately ≤20 μg/m^3^). Some possible explanations for high no-threshold levels above the world’s standards could be related to survival skills, immune response development, and sociocultural or religious practices in different geographical regions. For example, women in some countries in Northern Africa are required to wear veils for religious reasons and sometimes this would cover their mouth and nose, and this in turn may provide protection from inhaling dust particles suspended in the ambient air unlike their children who are not obligated to wear the same clothing and hence are at a higher rate of death from dust PM_2.5_ (3% and 1%, respectively). However, the veil-hypothesis has not been tested, and studies may be necessary to determine the same. The dust particles suspended in the ambient air are often there through natural occurrences (i.e., from wind and volcanoes) or manmade (i.e., from traveling vehicles and mining); and as the concentrations in the air decreases, the visibility improves and people may assume that the effect of fine particulates, which are capable of penetrating through the airways and being deposited in the alveolar region, has decreased, and, therefore, people will stay unprotected. Even at very low concentrations, PM_2.5_ has serious health impacts—actually, there is no threshold that has been identified below which it does not have health damage [33]. Recently, authors found that long-term exposure to low-concentrations of PM_2.5_ (≤6 μg/m^3^) is associated with mortality [6]. Therefore, more studies in the regions generally affected by dust is necessary to explain the findings of our study.

This study is the first of its kind, to the best of our knowledge, to explore the effect of different types of ambient PM_2.5_ on under-five and maternal mortality in Africa. Moreover, by using the global mortality data and the satellite information we were able to explore the entire region of Africa. In addition, we controlled for other population-based indicators, as well as temperature, which could confound the result of our study unlike other studies. Our results also sustained the findings of other studies that explored the adverse health effects of PM_2.5_ exposure. Therefore, this study provides epidemiologic evidence and a point of reference to studies on no-threshold levels of PM_2.5_ in different regions of Africa. More evidence, however, is still necessary to confirm the results of our findings.

Furthermore, by using two different analytical techniques in our study, the GLMM and the GAMM, we provided an innovative approach for exploring the long-term effect of PM_2.5_ and, as shown in our results, both the parametric and nonparametric approaches almost yielded the same effect size. Our analysis of potential confounders (i.e., the unadjusted) also revealed that the under-five and maternal deaths in Africa may have been as a result tuberculosis, which is an airborne disease, with an increased rate of 11% and 37%, respectively. This needs to be explored further because tuberculosis kills both adults and children alike, with the severe form mostly common in young children [34]. Another significant aspect of our study was the derivation of spectral AOD to distinguish aerosol types and subsequently the yearly average levels of exposure, hence reducing the bias associated with our exposure.

One limitation of our study is the lack of other environmental confounders that directly influence the relationship between PM_2.5_ and mortality, such as heat waves and drought periods, as well as individual and country-level information such as exposure time to ambient environment, smoking, cooking fuel, socioeconomic status, and age distribution. Hence, we may not have obtained the true effect size. However, we were able to adjust for temperature and several other population-based confounders such as the total population, the urban population, the employed population, the number of deaths as result of AIDS and tuberculosis, the number of undernourished people, and the prevalence of anemia in pregnant women. Nevertheless, some authors have found that census-based socioeconomic status does not confound the sub-regional PM_2.5_ results [35]. Another important limitation is the inability to control for other specific pollutants in the same spatial environment such as sulfur dioxide (SO_2_), nitrogen dioxide (NO_2_), and ozone (O_3_). Finally, by using an all-cause mortality we were unable to establish the true causality. However, our dose-response curve revealed a relationship at some level that needs to be explored further.

## 5. Conclusions

In conclusion, we explored the association between different types of ambient PM_2.5_ and mortality in Africa. Our study found that different types of ambient PM_2.5_ were associated with under-five and maternal mortality above the exposure level that exceeds the WHO global standards, and hence further research and subsequently a review of no-threshold levels in each region is therefore necessary. The policy implication of our findings is that developing and implementing appropriate preventive and control measures for different types of PM_2.5_ in different regions is appropriate.

## Figures and Tables

**Table 1:** Descriptive statistics of long-term under-five and maternal deaths, aerosol optical depth (AOD), types of PM_2.5_ and population characteristics, by Africa’s sub-regions from 2000 to 2015.

Variables	Northern Africa	Southern Africa	Eastern Africa	Western Africa	Central Africa	*p*-Value ^a^
	Mean (*SD*)	Mean (*SD*)	Mean (*SD*)	Mean (*SD*)	Mean (*SD*)	
Under-5 deaths	24,064.0 (23,525.1)	51,981.9 (53,012.2)	63,287.3 (76,937.6)	98,918.9 (203,881)	63,762.6 (98,947.8)	<0.001
Maternal deaths	672.4 (511.8)	2309.6 (2334.5)	4289.9 (5251.0)	6116.3 (13,813.6)	4253.8 (6476.2)	<0.001
**Biomass**						
AOD, *τ*	0.34 (0.14)	0.16 (0.07)	0.31 (0.14)	0.36 (0.15)	0.42 (0.15)	<0.001
Jan.–Mar. PM_2.5_, μg/m^3^	28.57 (10.42)	19.09 (4.39)	26.64 (9.60)	30.72 (10.98)	34.15 (10.51)	<0.001
Jan.–Jun. PM_2.5_, μg/m^3^	29.83 (9.02)	18.33 (4.55)	27.80 (8.40)	30.62 (9.42)	33.63 (8.54)	<0.001
Jan.–Sep. PM_2.5_, μg/m^3^	29.63 (8.61)	18.29 (3.79)	27.29 (7.95)	29.74 (8.34)	32.72 (8.27)	<0.001
Jan.–Dec. PM_2.5_, μg/m^3^	27.82 (7.60)	18.24 (3.43)	26.19 (7.60)	28.58 (8.08)	31.63 (7.99)	<0.001
**Anthropogenic**						
AOD, *τ*	0.20 (0.08)	0.14 (0.06)	0.21 (0.10)	0.27 (0.13)	0.29 (0.12)	<0.001
Jan.–Mar. PM_2.5_, μg/m^3^	35.66 (8.94)	27.11 (5.85)	35.26 (10.90)	42.03 (13.81)	43.95 (14.45)	<0.001
Jan.–Jun. PM_2.5_, μg/m^3^	37.07 (8.65)	25.83 (5.06)	37.67 (11.59)	43.92 (16.45)	47.46 (15.78)	<0.001
Jan.–Sep. PM_2.5_, μg/m^3^	36.02 (7.83)	28.70 (6.90)	37.87 (10.18)	43.54 (13.60)	45.60 (13.26)	<0.001
Jan.–Dec. PM_2.5_, μg/m^3^	34.66 (7.59)	28.74 (6.13)	36.04 (9.62)	41.99 (12.87)	43.89 (12.25)	<0.001
**Dust**						
AOD, *τ*	0.24 (0.11)	0.16 (0.06)	0.26 (0.12)	0.31 (0.14)	0.34 (0.12)	<0.001
Jan.–Mar. PM_2.5_, μg/m^3^	22.76 (6.43)	17.93 (2.62)	22.87 (6.43)	26.59 (8.66)	28.40 (6.66)	<0.001
Jan.–Jun. PM_2.5_, μg/m^3^	23.38 (5.89)	17.37 (3.02)	24.19 (6.73)	27.08 (8.48)	29.67 (6.85)	<0.001
Jan.–Sep. PM_2.5_, μg/m^3^	23.02 (5.69)	18.32 (3.34)	23.99 (6.73)	26.61 (7.56)	28.70 (6.48)	<0.001
Jan.–Dec. PM_2.5_, μg/m^3^	22.19 (5.69)	18.23 (2.99)	23.22 (6.58)	25.88 (7.27)	27.55 (6.26)	<0.001
**Mixture**						
AOD, *τ*	0.25 (0.11)	0.17 (0.06)	0.26 (0.10)	0.32 (0.14)	0.35 (0.12)	<0.001
Jan.–Mar. PM_2.5_, μg/m^3^	39.22 (8.45)	35.53 (4.29)	40.13 (7.84)	44.58 (10.94)	45.88 (8.28)	<0.001
Jan.–Jun. PM_2.5_, μg/m^3^	40.39 (8.52)	34.80 (4.18)	42.22 (8.63)	45.53 (10.17)	47.59 (8.40)	<0.001
Jan.–Sep. PM_2.5_, μg/m^3^	40.14 (8.56)	36.63 (5.19)	42.19 (8.29)	45.65 (9.42)	47.10 (8.65)	<0.001
Jan.–Dec. PM_2.5_, μg/m^3^	39.15 (8.57)	36.73 (4.78)	40.96 (8.28)	44.20 (8.78)	45.60 (8.47)	<0.001
Total population, million	31.1 (25.4)	14.1 (13.6)	18.8 (21.5)	18.8 (34.4)	12.5 (17.8)	<0.001
Urban population, million	16.4 (10.8)	5.9 (8.1)	4.5 (4.4)	7.4 (14.6)	4.8 (7.2)	<0.001
Female population, %	49.73 (0.76)	50.71 (0.51)	50.17 (0.60)	50.19 (0.63)	50.08 (0.56)	0.045
Employed population, %	42.23 (4.87)	59.53 (13.04)	62.72 (16.47)	65.37 (7.74)	66.33 (9.35)	<0.001
AIDS death, thousand	65.5 (128.5)	56.4 (76.8)	64.4 (105.8)	19.7 (42.2)	14.2 (15.2)	<0.001
Tuberculosis death rate, per 100,000	6.64 (5.28)	48.09 (23.12)	35.00 (28.17)	37.93 (24.46)	46.72 (34.16)	<0.001
Undernourished population, million	0.74 (1.0)	2.7 (2.7)	4.8 (8.7)	2.0 (2.3)	1.2 (1.6)	0.129
Anemia in pregnancy, %	32.76 (3.14)	38.70 (7.93)	40.18 (7.73)	57.28 (6.54)	53.15 (6.18)	<0.001
Annual mean temperature, °C	22.20 (2.08)	21.16 (2.82)	25.27 (2.32)	27.04 (1.50)	24.94 (1.53)	<0.001

^a^ General linear regression was used to test for. *SD*: Standard deviation.

**Table 2:** Unadjusted incidence rate ratio (IRR) and 95% confidence interval (CI) of under-5 and maternal mortality in Africa.

Variable	Under-5 Deaths, Unadjusted	Maternal Deaths, Unadjusted
	GLMM, IRR (95% CI) ^b^	GLMM, IRR (95% CI) ^b^
**Type of PM_2.5_^a^**		
Biomass	1.02 (1.01, 1.03) ****	1.07 (1.03, 1.10) ****
Anthropogenic	0.99 (0.99, 1.01)	1.03 (1.01, 1.04) ****
Dust	1.03 (1.01, 1.06) **	0.91 (0.90, 0.92) ****
Mixture	1.01 (1.01, 1.02) ****	1.07 (1.06, 1.09) ****
**Sub-regions (ref: North)**		
South	1.74 (0.26, 11.53)	3.33 (0.42, 26.67)
East	1.39 (0.23, 8.35)	3.56 (0.49, 25.40)
West	2.48 (0.42, 14.76)	5.82 (0.82, 41.48)
Central	1.25 (0.18, 8.64)	3.11 (0.37, 25.95)
Year	0.66 (0.64, 0.68) ****	0.81 (0.79, 0.82) ****
Total population	0.83 (0.81, 0.85) ****	1.02 (0.97, 1.05)
Urban population	1.02 (1.00, 1.03) *	0.99 (0.95, 1.03)
Female population	0.97 (0.92, 1.04)	1.10 (0.98, 1.24)
Employed population	1.10 (1.06, 1.12) ****	1.36 (1.33, 1.37) ****
AIDS death	1.02 (0.99, 1.04)	0.84 (0.78, 0.90) ****
Tuberculosis death	1.11 (1.06, 1.15) ****	1.37 (1.25, 1.49) ****
Undernourished population	0.91 (0.90, 0.92) ****	0.78 (0.75, 0.81) ****
Anemia in pregnancy	1.01 (1.00, 1.03) *	1.03 (0.95, 1.12)
Annual mean temperature	0.98 (0.97, 1.01)	1.05 (0.96, 1.14)

^a^ In one unit increment of PM_2.5_ concentration; ^b^ Natural cubit spline smoothing; GLMM, Generalized linear mixed-effect model; IRR, Incidence rate ratio; CI, Confidence interval; * *p* ≤ 0.10; ** *p* ≤ 0.05; **** *p* ≤ 0.001.

**Table 3:** Adjusted incidence rate ratio (IRR) and 95% confidence interval (CI) of the generalized linear mixed-effect model of under-5 and maternal mortality by sub-regions of Africa.

Annual Mean PM_2.5_^a^	Under-5 Deaths, Adjusted Model		Maternal Deaths, Adjusted Model	
	GLMM IRR (95% CI) ^b,c^	GAMM Coef. (*Se*) ^b^	GLMM IRR (95% CI) ^b,c^	GAMM Coef. (*Se*) ^b^
**Overall**				
Biomass	0.99 (0.98, 1.02)	0.02 (0.003) ****	0.94 (0.88, 0.99) *	0.06 (0.008) ****
Anthropogenic	1.01 (1.01, 1.02) ****	0.003 (0.0003) ****	1.03 (1.01, 1.05) **	0.04 (0.002) ****
Dust	0.98 (0.97, 0.98) ****	−0.003 (0.0005) ****	0.93 (0.91, 0.95) ****	−0.08 (0.002) ****
Mixture	0.94 (0.94, 0.94) ****	−0.01 (0.004) ****	0.95 (0.92, 0.98) ***	0.004 (0.006)
***Sub-regions***				
**Northern Africa**				
Biomass	0.90 (0.86, 0.94) ****	−0.05 (0.006) ****	1.02 (0.72, 1.42)	−0.03 (0.07)
Anthropogenic	1.05 (1.02, 1.08) ***	0.04 (0.002) ****	1.10 (1.00, 1.20) *	0.07 (0.03) **
Dust	1.03 (1.01, 1.06) **	0.03 (0.006) ****	1.07 (0.96, 1.19)	0.07 (0.04)
Mixture	0.99 (0.96, 1.02)	0.01 (0.004) ***	1.01 (0.90, 1.12)	0.02 (0.01)
**Southern Africa**				
Biomass	1.03 (0.99, 1.05)	0.03 (0.003) ***	1.01 (0.93, 1.08)	0.03 (0.02)
Anthropogenic	0.99 (0.97, 1.01)	−0.03 (0.002) ***	1.04 (0.94, 1.06)	0.01 (0.02)
Dust	1.01 (0.99, 1.02)	0.006 (0.002) **	0.98 (0.93, 1.03)	−0.01 (0.02)
Mixture	0.99 (0.95, 0.99) **	0.002 (0.003)	0.96 (0.90, 1.03)	−0.01 (0.02)
**Eastern Africa**				
Biomass	0.98 (0.97, 0.99) ****	−0.02 (0.003) ****	0.95 (0.89, 1.01)	−0.08 (0.01) ****
Anthropogenic	0.99 (0.99, 1.01)	−0.01 (0.001) *	1.04 (1.02, 1.05) ****	0.02 (0.006) **
Dust	1.01 (0.99, 1.04)	−0.01 (0.001) ****	0.95 (0.93, 0.96) ****	−0.06 (0.01) ****
Mixture	1.00 (0.99, 1.05)	0.001 (0.001)	1.06 (1.02, 1.09) ***	0.04 (0.01) ***
**Western Africa**				
Biomass	1.02 (1.01, 1.04) **	−0.001 (0.003) *	0.97 (0.91, 1.03)	0.01 (0.01)
Anthropogenic	1.01 (0.99, 1.02)	0.002 (0.0004) ****	1.02 (0.98, 1.06)	0.03 (0.003) ****
Dust	1.01 (1.01, 1.01) **	−0.003 (0.0003) ****	0.94 (0.92, 0.97) ****	−0.05 (0.003) ****
Mixture	1.01 (1.00, 1.02) *	−0.005 (0.0003) ****	0.93 (0.90, 0.96) ****	−0.04 (0.002) ****
**Central Africa**				
Biomass	1.02 (1.02, 1.03) **	0.01 (0.002) ****	1.19 (1.15, 1.23) ****	0.04 (0.01) ****
Anthropogenic	0.95 (0.99, 0.99) **	−0.004 (0.01)	1.00 (0.99, 1.00)	−0.01 (0.01)
Dust	1.10 (1.09, 1.10) ****	−0.002 (0.0005) ****	0.96 (0.93, 0.99) **	−0.02 (0.01) **
Mixture	0.99 (0.99, 0.99) **	−0.001 (0.001)	1.00 (0.99, 1.01)	−0.01 (0.01)

^a^ In one unit increment of PM_2.5_ concentration; ^b^ Adjusted for country, year, total population, urban population, female population, employed population, HIV/AIDS death, tuberculosis death, undernourished population, and temperature; ^c^ Natural cubit spline smoothing; GLMM, Generalized linear mixed-effect model; IRR, Incidence rate ratio; CI, Confidence interval; GAMM, Generalized Additive Mixed Model; Coef., Coefficient; *Se*, Standard error; * *p* ≤ 0.10; ** *p* ≤ 0.05; *** *p* ≤ 0.01; **** *p* ≤ 0.001.

**Figure A2:**
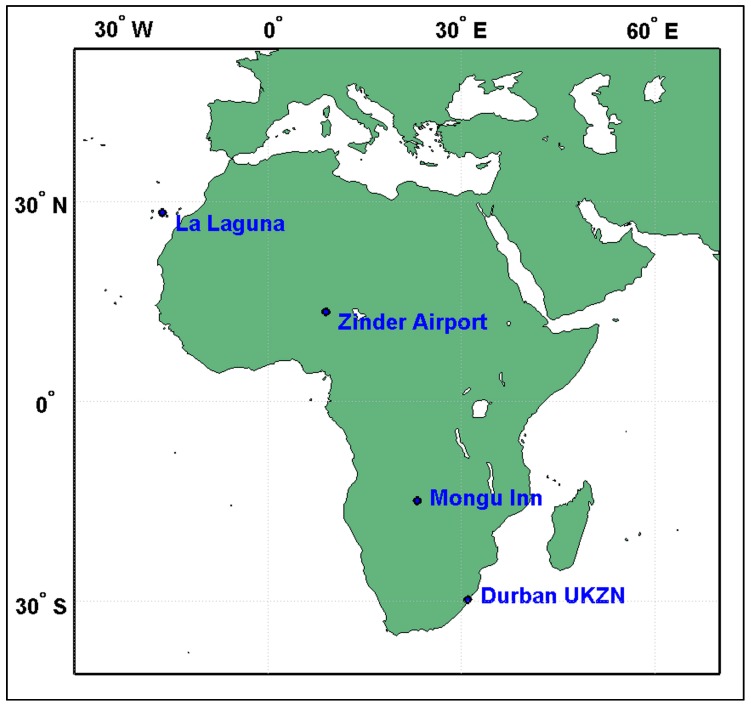
The distribution of the four ground-based measurements of AERONET sites include La Laguna (28.48° N, 16.32° W), Zinder Airport (13.78° N, 8.99° E), Mongu Inn (15.27° S, 23.13° E) and Durban UKZN (29.82° S, 30.94° E), from North Africa to South Africa.

**Figure A3:**
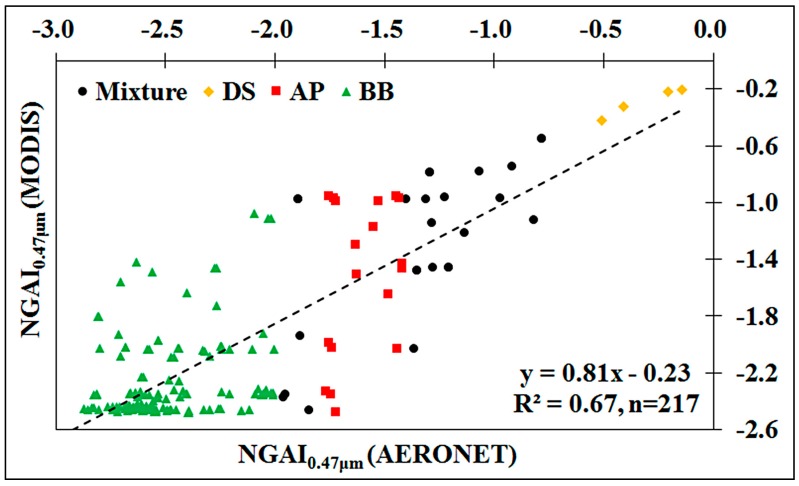
The comparison of aerosol categorization (217 cases) in terms of NGAI_0.47μm_ approach between MODIS AOD and ground-based measurement (AERONET) from January 2015 to March 2017. The colors in green, red, yellow and black represent BB, AP, DS and mixture aerosols respectively.

## Appendix A

For spectral characteristics, the optical property of aerosol is generally a function of wavelength, such as single scattering albedo (SSA) and AOD. The spectra derivatives of optical properties can be further linked to the characteristics of atmospheric particles in terms of particle size, scattering, and absorption processes. Association with the magnitude of the SSA value, the discrepancy in spectral behavior, could be a proxy for discriminating the type and mixed weight of present aerosols as the results illustrated in Figure A1. The PM_2.5_ concentration thus can be computed from the total AOD of each type (mixture included) by Equations (1) to (3) accordingly.

For the validation of aerosol categorization, the ground-based measurements from AERONET (Aerosol Robotic Network) are usually employed as the reference. Thus the NGAI values derived from the satellite AOD (MODIS) and the ground-based measurement (AERONET) were compared over Africa from January 2015 to March 2017 for this study. The four sites noted in Figure A2 are selected as representative stations for ground-based measurements from Northern to Southern Africa. The areas around La Laguna and Zinder Airport sites are frequently affected by DS, AP and mixed aerosols, while the BB, AP and mixed aerosols are usually observed in the region nearby Mongu Inn and Durban UKZN sites. A total 217 ground based measurements from 2015 to 2017 are carefully collected by considering the spatial resolution and acquired timing of MODIS AOD. The results displayed a linear relationship (*R*^2^ = 0.67) between the satellite and the ground-based measurements (see also Figure A3). It is noticed that the limitation of providing MODIS AOD products will happen when a serious aerosol event occurred, such as strong dust storms. That is the main reason why only few points can be classified as DS aerosol in Figure A3, especially in the Northern Africa region. Due to the difference of spatial coverage between the satellite observation (3 km) and the AERONET measurement (single point), some points that are far from the regression line. Overall, the results illustrated a good performance after compared with the ground based measurements, implying high practicality of NGAI approach for aerosol categorization.
